# Motherside care of the term neonate at birth

**DOI:** 10.1186/s40748-016-0034-9

**Published:** 2016-06-30

**Authors:** David Hutchon, Nick Bettles

**Affiliations:** Memorial Hospital, Darlington, UK; Delta Medical International Ltd, Sheffield, UK

**Keywords:** Motherside care, Neonatal resuscitation, Cord intact, Neonatal bonding, Mobile resuscitation trolley, Delayed cord clamping

## Abstract

The rationale for keeping the mother and her newborn together even when neonatal resuscitation is required is presented. The development of a customised mobile resuscitation trolley is detailed explaining how the resuscitation team can be provided with all the facilities of a standard resuscitation trolley to resuscitate the neonate at the mother’s side with an intact cord. Alternative low tech solutions which may be appropriate in low resource setting and with a low risk population are also described.

## Background

No mother wishes to be separated from her newborn baby immediately after birth. When resuscitation is considered necessary, the baby is removed from her and cared for on a resuscitation trolley at the side of the room with her baby usually out of her view. The distress this causes a mother is tolerated by her belief that the separation is unavoidable in order to provide essential care of her baby. For a number of reasons, many babies are needlessly separated from their mother at birth and require no active resuscitation by the time they reach the resuscitation trolley. The need for ventilatory assistance is the primary reason for moving the baby away from its mother but the majority of babies moved over to the resuscitation trolley do not require positive pressure ventilation (PPV). Effective PPV requires the neonate to be lying prone on a firm, flat but soft surface. The ventilatory equipment required needs to be immediately available and the area around the neonate accessible to the professional team. Effective thermal support for the neonate must be available, especially in a preterm birth.

All these facilities are standard in a hospital maternity unit resuscitation trolley but tradition and convenience has dictated that the designated area for resuscitation is not at the side of the mother but usually at the side of the room. Sometimes it is in a separate adjacent room.

The increased awareness of this separation by parents and the understanding of the importance of support and sometimes ventilation of the neonate while the placental circulation closes down naturally after birth, has led to a review of these arrangements [1]. The potential harm of early cord clamping has become widely recognised [2]. It is hard to argue that the potential harm of early cord clamping should be avoided in healthy neonates but then impose the risk of harm on the baby that is compromised by hypoxia during labour [3]. Can the facilities required by the professionals caring for the neonate be provided right by the side of the mother so that the neonate remains close enough for the cord circulation to remain potentially intact, and for her to be able to see and touch her baby? This has been a challenge which was first addressed in 2007.

In 2007 Van Rheenen was the first to point out that ventilation of the apnoeic neonate can be carried out with a bag and mask while the baby lies between its mother’s legs with the umbilical cord intact, and allowing delayed cord clamping for at least one minute [4]. Around the same time a feasibility study showed that with minor modifications, a standard resuscitation trolley could be brought close enough to the side of the mother to allow resuscitation with the cord intact [5].

## Modified standard resuscitation equipment

Bringing up the standard resuscitation equipment to the side of the mother is the most obvious solution and with minor modifications was shown to be practical. However there were limitations using equipment which was designed to remain at the side of the room. The procedure to bring the equipment up to the mother was clumsy so that in an acute situation it was not always possible to get the equipment close enough in sufficient time. Motherside resuscitation with the cord intact required considerable co-operation between the obstetricians and the neonatologists [6]. Some standard equipment may be more suitable than others but clearly a customised solution was an obvious way forwards. The first attempt to achieve a customised resuscitation trolley was realised with the concept prototype BASICS (**B**edside **A**ssessment **S**tabilisation **I**mmediate **C**ardiorespiratory **S**upport) trolley in 2010 [6].

## The customised resuscitation trolley

When considering the design of a customised resuscitation trolley (the LifeStart system (http://www.inditherm.com/medical/neonatal-resuscitation-lifestart/) there were three broad challenges: meeting the clinical needs; compliance with the medical device regulations; and commercial viability. The BASICS trolley, had identified many of the challenges and had proven viability in principle. However, this was not in a suitable format for medical device compliance and had not overcome some of the practical and aesthetic issues that would allow market acceptance.

In respect of the clinical application the key challenges were the need for a compact design due to space constraints, provision of all the facilities needed for resuscitation, and the ability to position the unit appropriately. There were however significant challenges in overcoming the conflicts between the design characteristics required to meet the clinical needs and the constraints imposed by the medical device regulations in respect of stability. These challenges were compounded by the need to consider stability in worst case conditions, considering all additional resuscitation equipment that might be mounted on the unit.

The need to position the unit in the right place in confined space requires very good mobility. In the original concept this was achieved by having a top which could swivel into position, however this required two handed operation. Further to this, for routine clinical use there was a need to provide a locking mechanism to prevent unwanted movement of the top after positioning. Whilst an elegant solution was designed, this added significant cost and complexity. It was ascertained that having the whole unit very manoeuvrable, with a fixed top, resulted in a much more practical solution that was easier to use.

In order to meet the needs of different types of delivery (vaginal, assisted, caesarean section) the unit had to have a wide range of heights for the resuscitation platform. The lowest position was defined by assisted deliveries where the unit needed to fit under the mother’s legs in lithotomy position. The highest was for caesarean section where the position was typically defined by the obstetrician’s preferred operating height. The original concept had a mechanical height adjustment, but this was again constrained in practical terms by the need for interlock mechanisms, requiring two handed operation and making precise adjustments difficult. It was therefore decided that for practical and commercial reasons an electrically operated height adjustment system was needed. Such an actuator with good stability, smooth, quiet operation, and wide adjustment range had already been identified and proved ideal for the purpose.

It is a requirement of the medical device standards that all electrical equipment must remain safe in any single fault condition. This adds a further challenge and in all the above design processes it was necessary to evaluate the implications of any single failure and provide a fail-safe solution.

The challenges of taking a concept design to a commercially viable product are considerable. The above outlines some of the design and compliance issues, however there are more general and subjective matters in relation to producing a product that will be accepted in the market. In this case one of the considerations was how to provide the resuscitation facilities such as suction, air/oxygen blending and positive end expiratory pressure (PEEP) ventilation. Every department will have their preferences for such devices and so the decision was made to provide standard medical mounting rails so that users could define their own configuration. This removes objections to third party devices and vastly simplifies the medical device approval process.

The dependence on piped supplies and electrical power did present a challenge in a significant number of cases at initial presentation of the product. However it was found that once users had become familiar with the system and implemented local protocols for its use, the issue tended to disappear. It is clear that in most cases the solution is both practical and convenient, although where piped medical air supply is not available the need to have a large cylinder at the side of the room is not ideal. Modification of the facilities and design of the delivery room may sometimes be just as important as the design of the equipment itself.

The product was brought to market in a very short timescale, largely due to the close collaboration between the company and the original clinical group, with a fast iterative design process. It is being used now in a number of UK units and at least one in the USA [7].

This approach has been appreciated by mothers who are able to see, and sometimes touch, for the first time, their newborn child (https://www.youtube.com/watch?v=TU0f8a3Cizo). The neonate is reassured by being able to continue to hear the familiar voice of its mother and father [8]. The trolley has been well accepted by clinicians [9].

## Low tech solutions

At the majority of births throughout the world there are no sophisticated facilities to resuscitate the neonate at birth. The Helping Babies Breathe program is widespread teaching the use of neonatal ventilation with an Ambubag and mask (http://www.helpingbabiesbreathe.org/). This is usually all that is necessary to start a baby breathing and the proposal by van Rheenen [4] to provide ventilation of the baby with the cord intact while it lies between the legs of the mother on a clean flat surface on the floor is the obvious low tech solution. Once breathing is well established the neonate can be held on the mother’s lap and breast feeding initiated.

## Low risk midwifery births

At the vast majority of low risk midwifery births the neonate requires no assistance during transition. The mother can hold her baby on her lap, skin to skin, covered with a warmed towel and there is the opportunity for breast suckling to commence. However the midwife does need to be prepared for the rare occasion when, without warning, the neonate is in poor condition and does not commence breathing [10]. The midwife may sometimes be the sole carer for both mother and baby. A readily available Ambubag and mask allows the midwife to provide PPV with the neonate lying between the mother’s legs on the delivery bed. It should be remembered that the neonate is still receiving oxygenated blood from the placenta for at least 90 s and this may be sufficient to prevent significant hypoxia by the apnoeic newborn [11]. Sometimes with stimulation alone the baby will make ventilatory efforts.

## Research and clinical practice

Such a major change in clinical practice does not happen overnight and the change is dependent partly upon demand by the parents and partly on the agreement that early cord clamping is harmful [12]. The neonatal resuscitation guidelines are neutral on ventilation before the cord is clamped although they do state that ventilation is a priority [13]. The WHO goes further and recommends ventilation before clamping and cutting the cord in the apnoeic neonate, provided there is sufficient skill and experience available to do this effectively [14].

As more evidence emerges and guidelines support delayed cord clamping, research specifically into resuscitation with the cord intact at the side of the mother will become ethically impossible and the confirmation will have to depend on cohort studies.

Co-operation between obstetricians, midwives and neonatologists has never been more important than in providing care of the neonate at the side of the mother. The procedure needs to be well rehearsed if it is to be effective. On the other hand it is not complex technology and good team work and preparation is all that is required for success. With an intact placental circulation providing a volume of oxygenated blood the majority of neonates should respond much more readily at resuscitation than the neonate with hypoxia and a potentially severe hypovolaemia, with fewer babies actually requiring resuscitation [15] .

With new resuscitation equipment now available the investigation and understanding of the haemodynamic changes at birth during delayed cord is simplified [7]. The equipment also allows the investigation of compromised neonates who require ventilatory assistance. Without this equipment and the ability to offer effective ventilation in both arms of a randomised controlled trial of resuscitation, a valid comparison of the traditional approach and resuscitation with an intact placental circulation is made impossible.

## Conclusion

Separation of the vast majority of babies from their mothers at birth is no longer acceptable. The common justification for separation at birth is the need for resuscitation. This can however be readily provided at the side of the mother with the placental and cord circulation intact using specially designed equipment. Research is needed to demonstrate the advantages for the baby and mother.

## Abbreviations

PPV, positive pressure ventilation; PEEP, peek end expiratory pressure; WHO, World Health Organisation
